# Colonization Density of the Upper Respiratory Tract as a Predictor of Pneumonia—*Haemophilus influenzae*, *Moraxella catarrhalis*, *Staphylococcus aureus*, and *Pneumocystis jirovecii*

**DOI:** 10.1093/cid/cix104

**Published:** 2017-05-27

**Authors:** Daniel E. Park, Henry C. Baggett, Stephen R. C. Howie, Qiyuan Shi, Nora L. Watson, W. Abdullah Brooks, Maria Deloria Knoll, Laura L. Hammitt, Karen L. Kotloff, Orin S. Levine, Shabir A. Madhi, David R. Murdoch, Katherine L. O’Brien, J. Anthony G. Scott, Donald M. Thea, Dilruba Ahmed, Martin Antonio, Vicky L. Baillie, Andrea N. DeLuca, Amanda J. Driscoll, Wei Fu, Caroline W. Gitahi, Emmanuel Olutunde, Melissa M. Higdon, Lokman Hossain, Ruth A. Karron, Abdoul Aziz Maiga, Susan A. Maloney, David P. Moore, Susan C. Morpeth, John Mwaba, Musaku Mwenechanya, Christine Prosperi, Mamadou Sylla, Somsak Thamthitiwat, Scott L. Zeger, Daniel R. Feikin, for the PERCH Study Group, Katherine L. O’Brien, Orin S. Levine, Maria Deloria Knoll, Daniel R. Feikin, Andrea N. DeLuca, Amanda J. Driscoll, Nicholas Fancourt, Wei Fu, Laura L. Hammitt, Melissa M. Higdon, E. Wangeci Kagucia, Ruth A. Karron, Mengying Li, Daniel E. Park, Christine Prosperi, Zhenke Wu, Scott L. Zeger, Nora L. Watson, Jane Crawley, David R. Murdoch, W. Abdullah Brooks, Hubert P. Endtz, Khalequ Zaman, Doli Goswami, Lokman Hossain, Yasmin Jahan, Hasan Ashraf, Stephen R. C. Howie, Bernard E. Ebruke, Martin Antonio, Jessica McLellan, Eunice Machuka, Arifin Shamsul, Syed M.A. Zaman, Grant Mackenzie, J. Anthony G. Scott, Juliet O. Awori, Susan C. Morpeth, Alice Kamau, Sidi Kazungu, Micah Silaba Ominde, Karen L. Kotloff, Milagritos D. Tapia, Samba O. Sow, Mamadou Sylla, Boubou Tamboura, Uma Onwuchekwa, Nana Kourouma, Aliou Toure, Shabir A. Madhi, David P. Moore, Peter V. Adrian, Vicky L. Baillie, Locadiah Kuwanda, Azwifarwi Mudau, Michelle J. Groome, Nasreen Mahomed, Henry C. Baggett, Somsak Thamthitiwat, Susan A. Maloney, Charatdao Bunthi, Julia Rhodes, Pongpun Sawatwong, Pasakorn Akarasewi, Donald M. Thea, Lawrence Mwananyanda, James Chipeta, Phil Seidenberg, James Mwansa, Somwe wa Somwe, Geoffrey Kwenda, Trevor P. Anderson, Joanne Mitchell

**Affiliations:** 1 Department of International Health, International Vaccine Access Center, Johns Hopkins Bloomberg School of Public Health, Baltimore, Maryland;; 2 Milken Institute School of Public Health, Department of Epidemiology and Biostatistics, George Washington University, Washington, District of Columbia;; 3 Global Disease Detection Center, Thailand Ministry of Public Health–US Centers for Disease Control and Prevention Collaboration, Nonthaburi;; 4 Division of Global Health Protection, Center for Global Health, Centers for Disease Control and Prevention, Atlanta, Georgia;; 5 Medical Research Council Unit, Basse, The Gambia;; 6 Department of Paediatrics, University of Auckland, and; 7 Centre for International Health, University of Otago, Dunedin, New Zealand;; 8 Emmes Corporation, Rockville, Maryland;; 9 Department of International Health, Johns Hopkins Bloomberg School of Public Health, Baltimore, Maryland;; 10 International Centre for Diarrhoeal Disease Research, Bangladesh (icddr,b), Dhaka and Matlab;; 11 Kenya Medical Research Institute–Wellcome Trust Research Programme, Kilifi;; 12 Division of Infectious Disease and Tropical Pediatrics, Department of Pediatrics, Center for Vaccine Development, Institute of Global Health, University of Maryland School of Medicine, Baltimore;; 13 Bill & Melinda Gates Foundation, Seattle, Washington;; 14 Medical Research Council, Respiratory and Meningeal Pathogens Research Unit, and; 15 Department of Science and Technology/National Research Foundation, Vaccine Preventable Diseases Unit, University of the Witwatersrand, Johannesburg, South Africa;; 16 Department of Pathology, University of Otago, and; 17 Microbiology Unit, Canterbury Health Laboratories, Christchurch, New Zealand;; 18 Department of Infectious Disease Epidemiology, London School of Hygiene & Tropical Medicine, United Kingdom;; 19 Center for Global Health and Development, Boston University School of Public Health, Massachusetts;; 20 Department of Pathogen Molecular Biology, London School of Hygiene & Tropical Medicine, and; 21 Microbiology and Infection Unit, Warwick Medical School, University of Warwick, Coventry, United Kingdom;; 22 Department of Epidemiology, Johns Hopkins Bloomberg School of Public Health,; 23 Department of Rheumatology, Johns Hopkins School of Medicine, and; 24 Department of International Health, Center for Immunization Research, Johns Hopkins Bloomberg School of Public Health, Baltimore, Maryland;; 25 Centre pour le Développement des Vaccins (CVD-Mali), Bamako;; 26 Division of Global HIV and Tuberculosis, Center for Global Health, Centers for Disease Control and Prevention, Atlanta, Georgia;; 27 Department of Paediatrics and Child Health, Chris Hani Baragwanath Academic Hospital and University of the Witwatersrand, Johannesburg, South Africa;; 28 Microbiology Laboratory, Middlemore Hospital, Counties Manukau District Health Board, Auckland, New Zealand;; 29 Department of Pathology and Microbiology, University Teaching Hospital,; 30 Zambia Center for Applied Health Research and Development, and; 31 Department of Pediatrics, University Teaching Hospital, Lusaka, Zambia;; 32 Department of Biostatistics, Johns Hopkins Bloomberg School of Public Health, Baltimore, Maryland, and; 33 Division of Viral Diseases, National Center for Immunizations and Respiratory Diseases, Centers for Disease Control and Prevention, Atlanta, Georgia; 34 Johns Hopkins Bloomberg School of Public Health, Baltimore, Maryland; 35 Emmes Corporation, Rockville, Maryland; 36 Nuffield Department of Clinical Medicine, University of Oxford, United Kingdom; 37 University of Otago, Christchurch, New Zealand; 38 icddr,b, Dhaka and Matlab, Bangladesh; 39 Medical Research Council, Basse, The Gambia; 40 KEMRI–Wellcome Trust Research Programme, Kilifi, Kenya; 41 Division of Infectious Disease and Tropical Pediatrics, Department of Pediatrics, Center for Vaccine Development, Institute of Global Health, University of Maryland School of Medicine, Baltimore, Maryland and Centre pour le Développement des Vaccins (CVD-Mali), Bamako, Mali; 42 Respiratory and Meningeal Pathogens Research Unit, University of the Witwatersrand, Johannesburg, South Africa; 43 Thailand Ministry of Public Health–US CDC Collaboration, Nonthaburi, Thailand; 44 Boston University School of Public Health, Boston, Massachusetts and University Teaching Hospital, Lusaka, Zambia; 45 Canterbury Health Laboratory, Christchurch, New Zealand

**Keywords:** pneumonia, colonization density, PERCH.

## Abstract

**Background.:**

There is limited information on the association between colonization density of upper respiratory tract colonizers and pathogen-specific pneumonia. We assessed this association for *Haemophilus influenzae*, *Moraxella catarrhalis*, *Staphylococcus aureus*, and *Pneumocystis jirovecii*.

**Methods.:**

In 7 low- and middle-income countries, nasopharyngeal/oropharyngeal swabs from children with severe pneumonia and age-frequency matched community controls were tested using quantitative polymerase chain reaction (PCR). Differences in median colonization density were evaluated using the Wilcoxon rank-sum test. Density cutoffs were determined using receiver operating characteristic curves. Cases with a pathogen identified from lung aspirate culture or PCR, pleural fluid culture or PCR, blood culture, and immunofluorescence for *P. jirovecii* defined microbiologically confirmed cases for the given pathogens.

**Results.:**

Higher densities of *H. influenzae* were observed in both microbiologically confirmed cases and chest radiograph (CXR)–positive cases compared to controls. *Staphylococcus aureus* and *P. jirovecii* had higher densities in CXR-positive cases vs controls. A 5.9 log_10_ copies/mL density cutoff for *H. influenzae* yielded 86% sensitivity and 77% specificity for detecting microbiologically confirmed cases; however, densities overlapped between cases and controls and positive predictive values were poor (<3%). Informative density cutoffs were not found for *S. aureus* and *M. catarrhalis*, and a lack of confirmed case data limited the cutoff identification for *P. jirovecii*.

**Conclusions.:**

There is evidence for an association between *H. influenzae* colonization density and *H. influenzae*–confirmed pneumonia in children; the association may be particularly informative in epidemiologic studies. Colonization densities of *M. catarrhalis*, *S. aureus*, and *P. jirovecii* are unlikely to be of diagnostic value in clinical settings.

Many pneumonia pathogens can also be upper respiratory tract (URT) colonizers, including *Streptococcus pneumoniae*, *Haemophilus influenzae*, *Staphylococcus aureus*, *Moraxella catarrhalis*, and *Pneumocystis jirovecii* [1–6]. Obtaining specimens from the site of infection remains challenging, as direct lung aspiration is rarely done, and blood cultures are insensitive and often unavailable in areas of highest pneumonia burden [7, 8]. In many settings, the high frequency of URT colonization with these potential pathogens in healthy children undermines the application of qualitative diagnostic tests, such as polymerase chain reaction (PCR), to ascribe etiology [9].

Previous studies have suggested that children with pneumonia may have higher pathogen density in the URT compared to children without pneumonia, though there is heterogeneity by study and pathogen [1, 4, 10–19]. We set out to determine if density of URT colonizers predicted pathogen-specific infections among pneumonia cases in the Pneumonia Etiology Research for Child Health (PERCH) study. Provided differences in densities between cases and controls, we evaluated whether pathogen densities offer any value in pneumonia diagnostic algorithms, or provide information beyond presence or absence of positivity alone in analytic models such as the PERCH integrated analysis [20]. The density evaluation for *S. pneumoniae* is reported elsewhere [21].

## METHODS

The PERCH study is a multicountry, standardized evaluation of the etiologic agents causing severe and very severe pneumonia among children in sites in 7 low- and middle-income countries: Dhaka and Matlab, Bangladesh; Basse, The Gambia; Kilifi, Kenya; Bamako, Mali; Soweto, South Africa; Nakhon Phanom and Sa Kaeo, Thailand; and Lusaka, Zambia. *Haemophilus influenzae* type b vaccine was used routinely at all sites except Thailand, while South Africa, The Gambia, Mali, and Kenya used pneumococcal conjugate vaccine throughout the duration of the study [22]. PERCH followed a standardized protocol for enrollment, specimen collection, and laboratory testing [23].

### Case and Community Control Selection and Clinical Evaluation

Identification and selection of cases and controls have been described previously [24]. In brief, we enrolled hospitalized patients aged 1– 59 months with World Health Organization (WHO)–defined severe or very severe pneumonia and age-frequency matched community controls. Severe pneumonia was defined as having cough or difficulty breathing and lower chest wall indrawing; very severe pneumonia was defined as cough or difficulty breathing and at least 1 of the following: central cyanosis, difficulty breastfeeding/drinking, vomiting everything, convulsions, lethargy, unconsciousness, or head nodding [25]. Within this case definition we further defined radiographic pneumonia as consolidation or any other infiltrate on chest radiograph (CXR positive) as interpreted by a panel of trained CXR readers [26, 27]. Microbiologically confirmed cases were those with identification of the respective pathogen by PCR from lung aspirate or pleural fluid; bacterial culture from lung aspirate, pleural fluid, or blood; or *P. jirovecii* by induced sputum, pleural fluid, or lung aspirate immunofluorescence or toluidine blue staining [28]. Antibiotic pretreatment was defined by having either positive serum bioassay or clinician report of antibiotics administered prior to specimen collection on the day of admission.

### Specimen Collection

A flocked nasopharyngeal (NP) swab (flexible minitip, Copan) and a rayon oropharyngeal (OP) swab (Fisher Scientific), transported in universal transport media (Copan) and processed within 24 hours of collection were used for URT pathogen detection by PCR. Blood was collected for culture. Pleural fluid specimens were collected as clinically indicated. Lung aspirates were collected from a subset of cases in The Gambia, South Africa, Mali, and Bangladesh.

### Laboratory Testing

PERCH employed conventional and molecular diagnostic techniques for the identification of potential pathogens, as described elsewhere [8, 28–31]. In brief, total nucleic acid extraction was performed on respiratory specimens using the NucliSens easyMAG system (bioMérieux, Marcy l’Etoile, France). Four hundred microliters of each respiratory specimen was eluted to a final volume of 60–110 μL nucleic acid.

Respiratory specimens (including lung aspirate and pleural fluid specimens) were evaluated using the Fast Track Diagnostics Respiratory Pathogens 33 test (FTD Resp-33) (Fast-track Diagnostics, Sliema, Malta), a 33-target, 8-multiplex real-time quantitative PCR platform for the detection of selected viruses and the following bacteria and fungi: *P. jirovecii*; *Mycoplasma pneumoniae*; *Chlamydophila pneumoniae*; *S. pneumoniae*; *H. influenzae* type b; *H. influenzae* species; *S. aureus*; *M. catarrhalis*; *Bordetella pertussis*; *Klebsiella pneumoniae*; and *Salmonella* species. Standard curves for quantification were generated using 10-fold serial dilutions of plasmid standards provided by FTD approximately every 3 months and were used to calculate pathogen density (log_10_ copies/mL) from the sample cycle threshold values; standards were only available for the linear range of detection of the assay from 4.0 to 8.0 log_10_ copies/mL. Additionally, induced sputum, pleural fluid, and lung aspirate specimens were tested for *P. jirovecii* by immunofluorescence (South Africa) and toluidine blue staining (Zambia).

Blood cultures were incubated using automated systems (BacT/ALERT in South Africa, Thailand, and Bangladesh; BACTEC at all other sites). Organisms were identified according to standard microbiological methods.

### Statistical Analysis

We made comparisons between community controls and both microbiologically confirmed pneumonia cases and radiographic pneumonia cases. Microbiologically confirmed cases were not restricted to children with abnormal findings on CXR in order to include children who died before a radiograph was taken or had not developed a positive finding at time of initial radiograph. Human immunodeficiency virus (HIV)–infected cases were included in a supplemental analysis for *P. jirovecii* but excluded from all other analyses.

Among children positive for each organism, mean and median colonization densities were compared using Wilcoxon rank-sum tests. Potential covariates of colonization density were evaluated, including site, age, sex, vaccination status, and prior antibiotic administration [32]. Total bacterial load (across all bacteria tested for by FTD Resp-33) was compared between cases and controls, in addition to proportional pathogen densities comparing proportional contributions of a given organism to the total bacterial load. Receiver operating characteristic (ROC) curves and the corresponding area under the curve (AUC) were generated to investigate the performance of absolute density in determining case status among microbiologically confirmed cases and community controls including children without detection of the given pathogen in the URT by PCR, and also between radiographic pneumonia cases and community controls positive for each organism in the URT by PCR. The Youden index was calculated to determine the best-performing cutoffs to differentiate cases and community controls [33]. To guard against bias in the estimates of sensitivity due to having a small number of confirmed cases for each potential pathogen, the Youden index was calculated using leave-one-out cross-validation when sample sizes were sufficient [34]. Positive and negative predictive values associated with each cutoff were calculated comparing microbiologically confirmed cases as the gold standard against all HIV-uninfected cases.

All analyses were performed using SAS software version 9.4 (SAS Institute, Cary, North Carolina) and R Statistical Software 3.2.1 (R Foundation for Statistical Computing, Vienna, Austria). *P* values are 2 sided.

### Ethical Considerations

The PERCH study protocol was approved by the institutional review board or ethical review committee at each of the study site institutions and at the Johns Hopkins Bloomberg School of Public Health. Parents or guardians of all participants provided written informed consent.

## RESULTS

Of the 4232 cases enrolled in the PERCH study, 4139 cases had available NP/OP PCR results; of those, 239 additional cases were excluded for being HIV infected, resulting in 3900 cases. Among the 3900 cases, 52 (1.3%) were microbiologically confirmed for at least 1 organism of interest, 1733 (44.4%) had an abnormal CXR, and 1700 (43.6%) had an abnormal CXR without microbiologic confirmation of *H. influenzae*, *M. catarrhalis*, *S. aureus*, or *P. jirovecii* (Table 1). Site sample sizes for CXR-positive cases varied from 425 cases in South Africa to 97 cases in Thailand. From 5118 enrolled HIV-negative controls, 4986 (97.4%) had available NP/OP PCR results. Compared to controls, cases tended to be younger and were more likely to have had antibiotics prior to their NP/OP sample collection.

**Table 1.** Characteristics of Children Included in Analysis of Quantification^a^

Characteristic		Microbiologically Confirmed Cases^b^ (n = 52)	CXR-Positive Cases^c^ (n = 1700)	Controls (n = 4986)
Site	Kenya	4 (7.7)	278 (16.4)	855 (17.1)
	The Gambia	8 (15.4)	267 (15.7)	624 (12.5)
	Mali	14 (26.9)	229 (13.5)	724 (14.5)
	Zambia	11 (21.2)	185 (10.9)	535 (10.7)
	South Africa	13 (25.0)	425 (25.0)	823 (16.5)
	Thailand	2 (3.8)	97 (5.7)	657 (13.2)
	Bangladesh	0 (0)	219 (12.8)	768 (15.4)
Age	1-5 mo	24 (46.2)	667 (39.2)	1555 (31.2)
	6-11 mo	11 (21.2)	409 (24.1)	1187 (23.8)
	12-23 mo	11 (21.2)	414 (24.4)	1235 (24.8)
	24-59 mo	6 (11.5)	210 (12.4)	1009 (20.2)
Sex	Female	32 (61.5)	736 (43.3)	2477 (49.7)
Antibiotics^d^	Prior to NP/OP swab collection	22 (42.3)	791 (46.5)	84 (1.7)
No. of bacteria detected^e^	0	…	75 (4.4)	141 (2.8)
	1	2 (3.8)	197 (11.6)	486 (9.7)
	2	9 (17.3)	376 (22.1)	1141 (22.9)
	≥3	40 (76.9)	1033 (60.8)	3195 (64.1)

*P* value <.05 for case-control group comparisons of overall site, overall age, sex, prior antibiotics, and number of bacteria detected, obtained from χ^2^ test.

Data are presented as No. (%).

Abbreviations: CXR, chest radiograph; NP/OP, nasopharyngeal/oropharyngeal.^a^Children with available NP/OP polymerase chain reaction results.

^b^Microbiologically confirmed for *Haemophilus influenzae*, *Moraxella catarrhalis*, *Staphylococcus aureus*, or *Pneumocystis jirovecii.*

^c^CXR positive defined as having radiographic evidence of pneumonia (consolidation and/or other infiltrate), excluding microbiologically confirmed cases of *H. influenzae*, *M. catarrhalis*, *S. aureus*, or *P. jirovecii*.

^d^Administered antibiotics at the study facility prior to the collection of specimens (cases only), positive serum bioassay (cases and controls), received antibiotics at a referral facility (cases only), or received routine cotrimoxazole prophylaxis (cases and controls).

^e^Including *Bordetella pertussis*, *Chlamydophila pneumoniae*, *H. influenzae*, *M. catarrhalis*, *Mycoplasma pneumoniae*, *P. jirovecii*, *S. aureus*, *Salmonella* species, and *Streptococcus pneumoniae.*

### Prevalence and Density Comparisons Between Microbiologically Confirmed Cases, Radiographic Pneumonia Cases, and Community Controls

All 21 cases with microbiologically confirmed *H. influenzae* (8 type b, 9 nontypeable, 2 type a, 2 unknown) were NP/OP positive for *H. influenzae*. Among the 21 cases, 14 (66.7%) were CXR positive. Six confirmed *M. catarrhalis* cases were NP/OP positive, among which 4 (66.7%) were CXR positive. Fifteen confirmed *S. aureus* cases were NP/OP positive, among which 10 (66.7%) were CXR positive. One confirmed *P. jirovecii* case was NP/OP positive and was also CXR positive. Only *H. influenzae* was found at significantly higher median densities in microbiologically confirmed cases compared to controls (Table 2).

**Table 2.** Detection Prevalence and Median Pathogen Density (Copies/mL) From Nasopharyngeal/Oropharyngeal Swabs for Microbiologically Confirmed Cases, Non–Microbiologically Confirmed Radiographic Pneumonia Cases, Cases Microbiologically Confirmed for Another Pathogen, and Controls— All Sites

		A. Confirmed Cases^a^	B. CXR-Positive Cases (n = 1657)	C. Cases Confirmed for Other Pathogen^b^	D. All Controls (n = 4986)	Difference in Median Densities, *P* Value^c^		
Colonizer	Measure					A vs C	A vs D	B vs D
*Haemophilus influenzae*	NP/OP+, No. (%)^d^	21/21 (100)	949 (57.3)	76/121 (62.8)	2562 (51.5)			
	Median density (IQR)^e^	6.77 (6.00–7.12)	5.90 (5.00–6.66)	6.39 (5.53–7.08)	5.71 (4.89–6.35)	.19	<.001	<.001
*Moraxella catarrhalis*	NP/OP+, No. (%)^d^	6/7 (85.7)	1091 (65.8)	92/135 (68.1)	3694 (74.3)			
	Median density (IQR)^f^	6.01 (5.00–6.73)	5.50 (4.65–6.26)	6.15 (5.09–6.78)	5.59 (4.87–6.20)	.88	.44	.07
*Pneumocystis jirovecii*	NP/OP+, No. (%)^d^	1/2 (50.0)	149 (9.0)	10/140 (7.1)	382 (7.7)			
	Median density (IQR)^f^	4.01 (NA)	3.92 (3.08–4.73)	4.00 (2.14–5.95)	3.56 (3.02–4.10)	…	.44	<.001
*Staphylococcus aureus*	NP/OP+, No. (%)^d^	15/23 (65.2)	342 (20.6)	29/119 (24.5)	940 (18.9)			
	Median density (IQR)^f^	4.87 (3.87–5.64)	4.48 (3.43–5.53)	5.14 (4.46–5.85)	4.29 (3.42–5.17)	.43	.13	.05

Abbreviations: CXR, chest radiograph; IQR, interquartile range; NA, not applicable; NP/OP, nasopharyngeal/oropharyngeal.

^a^Detection of relevant pathogen from blood culture, lung aspirate culture/polymerase chain reaction (PCR), pleural fluid culture/PCR, pneumocystis immunofluorescence or staining.

^b^Microbiologically confirmed case for any other bacteria or virus.

^c^Comparing median densities using Wilcoxon rank-sum test.

^d^No. (%) positive in the NP/OP for organism among those with available results for that target.

^e^Log_10_ copies/mL, among all confirmed case positives including NP/OP PCR negatives counted as zero densities.

^f^Among those positive on NP/OP PCR.

**Figure 1.**
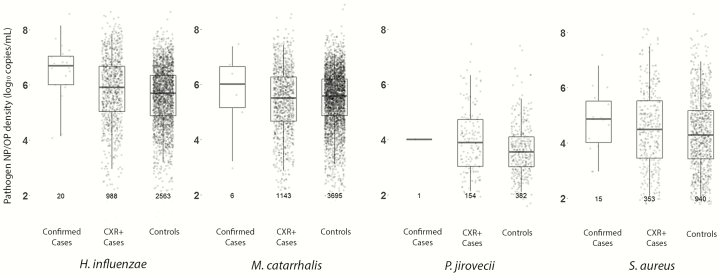
Median density in the nasopharynx/oropharynx (NP/OP) among microbiologically confirmed cases, radiographic pneumonia (chest radiograph positive [CXR+]) cases^a^, and controls, restricted to participants testing NP/OP positive for the given pathogen. Horizontal lines through boxes indicate group medians. Boxes extend to the 25th and 75th percentiles. Whiskers extend to minimum and maximum. ^a^Defined as having radiographic evidence of pneumonia (consolidation and/or other infiltrate), excluding confirmed cases.

Comparing non–microbiologically confirmed CXR-positive cases to controls, *H. influenzae* was more commonly detected in the NP/OP of cases vs controls (57.3% vs 51.5%, *P* < .001). Prevalence was similar between CXR-positive cases and controls for *S. aureus* and *P. jirovecii*. *Moraxella catarrhalis* detection was more common among controls than CXR-positive cases, even after adjusting for site and prior antibiotics (65.8% vs 74.3%, *P* ≤ .001). Median density (log_10_ copies/mL) was higher in CXR-positive cases compared to controls for *H. influenzae*, *S. aureus*, and *P. jirovecii*, while *M. catarrhalis* median density was similar in CXR-positive cases and controls. For all organisms, there was substantial overlap of the distribution of densities between microbiologically confirmed cases, CXR-positive cases, and controls (Figure 1).

Though densities varied by site, trends between microbiologically confirmed cases, CXR-positive cases, and controls remained and were consistent across age categories, WHO-defined severity categories, and sex (Supplementary Figure 2). Prior antibiotic use was associated with a lower density for *H. influenzae* (6.15 vs 5.64 log_10_ copies/mL) and *M. catarrhalis* (5.77 vs 5.21 log_10_ copies/mL) among CXR-positive cases (Supplementary Figure 3). There were similar rates of antibiotic use prior to NP/OP specimen collection between microbiologically confirmed cases and nonconfirmed CXR-positive cases. HIV-infected, CXR-positive cases had a higher median density compared to HIV-negative, CXR-positive cases for *P. jirovecii* (5.03 vs 3.78 log_10_ copies/mL) (Supplementary Figure 4).

### Total and Proportional Bacterial Density

Total bacterial density across all bacterial targets from our PCR panel was similar among consolidated pneumonia cases (5.90 log_10_ copies/mL), CXR-positive cases (5.81 log_10_ copies/mL), and controls (5.75 log_10_ copies/mL), and also similar among CXR-positive cases across age groups, adjusting for prior antibiotic use and site. Evaluating whether the proportion of total bacterial density attributable to specific organisms differed between case and control groups, median relative proportions of *H. influenzae* tended to be higher in *H. influenzae*–confirmed cases (41.6%) and CXR-positive cases (7.2%) compared with controls (1.2%). Conversely, median proportional densities of *M. catarrhalis* were higher in controls compared to CXR-positive cases (14.3% vs 5.0%). Among 100 randomly selected controls, 24% had *M. catarrhalis–*dominated proportional densities (≥50%) compared with 14%–16% of non–*M. catarrhalis* confirmed cases (Supplementary Figure 6). However, there was substantial overlap in the range of proportional densities between microbiologically confirmed cases, CXR-positive cases, and controls.

### Determination of Density Cutoffs

**Figure 2.**
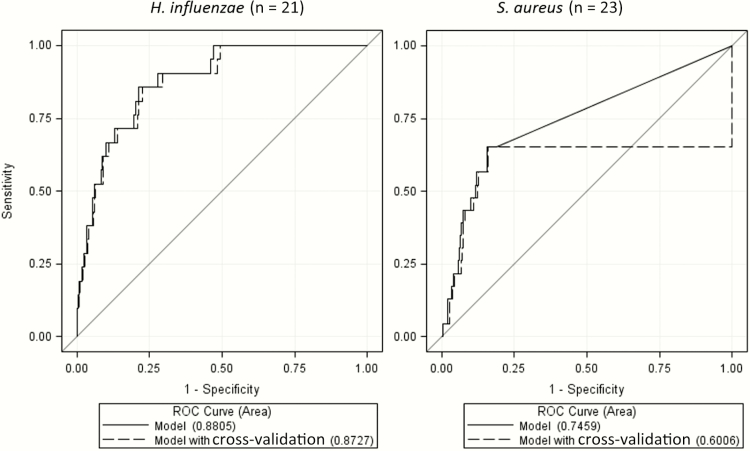
Receiver operating characteristic (ROC) curve and Youden index analysis for *Haemophilus influenzae* and *Staphylococcus aureus* confirmed cases compared with controls.

Using density in log_10_ copies/mL in microbiologically confirmed cases compared to controls, the ROC curve best-performing cutoff of 5.9 log_10_ copies/mL was identified for *H. influenzae* with a sensitivity of 86% and specificity of 77% (Figure 2 and Table 3). Though the *S. aureus* cutoff (3.0 log_10_ copies/mL) was moderately sensitive and specific, the *S. aureus* cutoff did not significantly improve sensitivity and specificity over qualitative analysis using simply presence or absence of specific organisms.

**Table 3.** Receiver Operating Characteristic Curve Nasopharyngeal/Oropharyngeal Cutoffs^a^ for Determining Case Status and Corresponding Area Under the Curve, Positive Proportion in Cases, and Negative Proportion in Controls by Pathogen

	Confirmed Cases vs Controls				NP/OP-Positive, CXR-Positive Cases^b^ vs Controls			
Colonizer	Best-Performing Cutoff (Log_10_ Copies/mL)	AUC	Proportion of Cases Above Cutoff	Proportion of Controls Below Cutoff	Best-Performing Cutoff (Log_10_ Copies/mL)	AUC	Proportion of Positive Cases Above Cutoff	Proportion of Positive Controls Below Cutoff
*Haemophilus influenzae*	5.92	0.87	0.86	0.77	…	…	…	…
*Moraxella catarrhalis*	…	…	…	…	4.99	0.50	0.34	0.71
*Pneumocystis jirovecii*	…	…	…	…	4.01	0.58	0.48	0.72
*Staphylococcus aureus*	2.97	0.6	0.65	0.84	…	…	…	…

Abbreviations: AUC, area under the curve; CXR, chest radiograph; NP/OP, nasopharyngeal/oropharyngeal.

^**a**^Cutoffs for confirmed cases calculated using the Youden index for *H. influenzae*, and *S.aureus* (with cross-validation). Confirmed cases defined as any detection from blood culture, lung aspirate culture/polymerase chain reaction (PCR), or pleural fluid culture/PCR. Cutoffs for CXR-positive cases calculated using the Youden index (with cross-validation) comparing CXR-positive cases to controls, excluding children who were negative by NP/OP PCR.

^b^Defined as having radiographic evidence of pneumonia (consolidation and/or other infiltrate).

Given the limited numbers of NP/OP-positive microbiologically confirmed *P. jirovecii* (n = 1) and *M. catarrhalis* (n = 6) cases, we explored the use of NP/OP positive, CXR-positive cases and controls to identify potential cutoffs for *P. jirovecii* (4.0 log_10_ copies/mL) and *M. catarrhalis* (5.0 log_10_ copies/mL) (Supplementary Figure 5). Densities among the limited number of microbiologically confirmed cases were consistent with these cutoffs, as 5 of 6 microbiologically confirmed *M. catarrhalis* cases were above the thresholds. The *P. jirovecii* cutoff demonstrated 48% sensitivity and 72% specificity for identifying *P. jirovecii*–positive CXR-positive cases (AUC = 0.58), among cases and controls positive for *P. jirovecii* (98% specificity including negatives). *Moraxella catarrhalis* cutoffs did not help differentiate CXR-positive cases from controls (AUC = 0.50).

### Positive and Negative Predictive Values Using Dichotomous Positivity and Density Cutoffs

Positive predictive values (PPVs) for identifying microbiologically confirmed cases from all cases using dichotomous URT positivity were low and improved marginally when density cutoffs were applied to *H. influenzae* (1.01% to 1.75%), *S. aureus* (1.81% to 2.14%), and *P. jirovecii* (0.89% to 1.67%), though the PPV is limited by lack of sensitivity in identifying microbiologically confirmed cases. Conversely, negative predictive values for identifying *H. influenzae*, *S. aureus*, and *P. jirovecii* microbiologically confirmed cases remained >99% for any detection in the URT and when utilizing the density cutoff.

### Clinical Characteristics Associated With Being Above Versus Below Optimized Cutoffs

Densities above the cutoffs (compared to low densities and negatives) were associated with hypoxemia (adjusted odds ratio [AOR], 1.22; 95% confidence interval [CI], 1.02–1.47), any CXR abnormality (AOR, 1.23; 95% CI, 1.05–1.45), and primary endpoint consolidation (AOR, 1.57; 95% CI, 1.28–1.92) for *H. influenzae*, adjusted for site, age, sex, and prior antibiotic use. Additionally, having a density above the cutoff was associated with case fatality for *P. jirovecii* (AOR, 4.5; 95% CI, 2.6–7.5).

## DISCUSSION

Colonization density of the URT with *H. influenzae* was significantly higher in microbiologically confirmed cases compared with community controls. Given the high prevalence of *H. influenzae* as a common colonizer, the improved specificity provided by the optimized cutoff of 5.9 log_10_ copies/mL helped delineate between common colonization and *H. influenzae*–mediated pneumonia. However, the overlapping density distributions between all case and control groups, and the low PPV limit the utility of density in individuals for clinical diagnosis. Our findings are similar to results from a study in adult patients in Denmark that yielded 90% sensitivity and 65% specificity using a cutoff at 5.0 log copies/mL [17]. Another study in Vietnamese children did not find an association between NP *H. influenzae* density and radiographic pneumonia; however, findings may have been limited by lack of a confirmed case group [12].

Colonization density of *P. jirovecii* may provide information toward differentiating carriage and pathogen-mediated pneumonia, although our evaluation was limited by a lack of microbiologically confirmed pneumocystis pneumonia (PCP) cases. A potential cutoff at 4.0 log_10_ copies/mL conferred 50% sensitivity and 72% specificity for radiographic pneumonia; however, the proportion of radiographic pneumonia cases with PCP-mediated pneumonia is unknown, and the PERCH process for identifying CXR abnormality may have been less sensitive for detecting radiographic features uniquely associated with PCP. The potential density cutoff is in line with other studies that have identified cutoffs between approximately 3 and 4.5 log copies/mL using clinically confirmed PCP patients, though we have not standardized the quantitative standards across these different studies [14–16]. Utilizing *P. jirovecii* detection from induced sputum PCR is widely accepted in clinical practice and would have expanded our sample of microbiologically confirmed cases [35, 36]. However, these diagnostic tests are typically carried out when pneumocystis pneumonia is clinically suspected; findings from the PERCH study complicate the utility of induced sputum PCR as a confirmatory diagnostic tool in settings where nearly all cases had an induced sputum specimen collected [37].

Previous studies have failed to identify an association between density and pathogen-confirmed pneumonia for *M. catarrhalis* and *S. aureus* [1]. While the optimized *S. aureus* cutoff was specific, the relatively high proportion of NP/OP negatives among the microbiologically confirmed cases precludes the identification of a highly sensitive and specific cutoff. It has been suggested that *Moraxella* and *Corynebacterium*/*Dolosigranulum*-dominated microbiota profiles confer stability and are protective against respiratory disease, while *Streptococcus*- and *Haemophilus*-dominated microbiota profiles enhance susceptibility to respiratory infections [38, 39]. The protective effect of *M. catarrhalis* is supported by our finding of both higher prevalence, proportional density, and absolute density in controls compared to radiographic pneumonia cases.

Though our analysis benefited from a large sample size enrolled from multiple heterogeneous study sites and comprehensive clinical and laboratory standardization, there were limitations. Despite our large overall sample size, the number of microbiologically confirmed cases was limited. However, leave-one-out cross validation and findings from comparisons of CXR-positive cases vs controls supported findings from microbiologically confirmed cases. While we were able to evaluate co-pathogen interactions and proportional densities between a select number of potential pathogens, a metagenomics or microbiome approach would be better suited to understand the contribution of individual pathogens in the context of the upper respiratory tract microbiome [40–45]. Furthermore, we were unable to establish temporality between higher densities in the NP/OP and subsequent development of pneumonia, as infection in the lung may lead to higher densities in the NP/OP. Longitudinal studies would be better suited to addressing the role of colonization density on the development of pneumonia. The study design did not control for prior antibiotic use, which was associated with lower densities for both *H. influenzae* and *M. catarrhalis*. However, because prior antibiotic use was more common among cases compared to controls, the bias was toward the null for the analyses. *Staphylococcus aureus* and *P. jirovecii* are unlikely to be significantly affected by first-line antibiotics, which was reflected in our findings [46]. Variability of density distributions by site was also observed; however, density trends between the microbiologically confirmed case, radiographic pneumonia case, and control groups within a site followed the overall trends.

While we have provided cutoffs optimized for sensitivity and specificity, the choice of a cutoff can be tailored for specific applications. Identifying a density cutoff that maximizes combined sensitivity and specificity provides additional information from the NP/OP specimen in the PERCH primary etiology analysis; however, the interpretation and application of these cutoffs may not be ideal for use as either a diagnostic or screening tool (which may prioritize specificity and sensitivity, respectively). Moreover, the PPV using the density cutoff remained low for *H. influenzae*, although the PPV was limited by poor sensitivity for identifying microbiologically confirmed cases and low prevalence. Regardless, it is likely impossible to identify a URT density cutoff for these colonizers that could be applied as a clinical gold standard because the densities overlap and span the PCR linear range in both cases and controls, precluding the identification of clear cutoffs that would independently guide clinical decisions.

We found evidence for the relationship between *H. influenzae* colonization density and *H. influenzae–*mediated pneumonia in children, and also a potential association between *P. jirovecii* colonization density and pathogen-specific pneumonia. The use of molecular diagnostics from URT specimens provides significant advantages in both sensitivity and speed over traditional culture diagnostics, but ascribing lung infection based on detection of colonization in the URT is challenging. Compared to using presence or absence of positivity, utilizing colonization density improves specificity of molecular diagnostics with small reductions in sensitivity and improves information from the URT in the context of population-level epidemiologic studies such as the PERCH integrated analysis, but remains suboptimal for use as a gold standard diagnostic in clinical settings at the individual case level.

## Supplementary Data

Supplementary materials are available at *Clinical Infectious Diseases* online. Consisting of data provided by the author to benefit the reader, the posted materials are not copyedited and are the sole responsibility of the author, so questions or comments should be addressed to the corresponding author.

## Supplementary Material

Supplemental Materials

## Notes

***Author contributions.*** D. E. P. led the analysis, interpreted results, and drafted the manuscript. Q. S. performed analyses and assisted with interpretation of results. D. R. F., H. C. B., and S. R. C. H. assisted with interpretation of results and drafting of the manuscript. O. S. L., K. L. O., D. R. F., D. R. M., M. D. K., L. L. H., H. C. B., W. A. B., S. R. C. H., K. K. L., S. A. M., J. A. G. S., D. M. T., and R. A. K. conceived and designed the study and supervised study conduct. D. A., M. A., V. L. B., A. N. D., A. J. D., W. F., C. W. G., E. O., M. M. H., L. H., A. A. M., S. M., D. P. M., S. C. M., J. W., M. M., C. P., M. M., and S. T. were involved in study conduct, data collection, and/or data management. S. Z. provided statistical guidance. All authors reviewed and approved the manuscript. D. E. P. had full access to the data and had final responsibility for the decision to submit for publication.

***Acknowledgments.*** We offer sincere thanks to the patients and families who participated in this study. We acknowledge members of the following group who contributed to the study design, conduct, and/or analysis phases of Pneumonia Etiology Research for Child Health (PERCH) (see Supplementary Materials for full list of names): Pneumonia Methods Working Group, PERCH Expert Group, PERCH Contributors, and the PERCH Chest Radiograph Reading Panel.

***PERCH Study Group.*** Johns Hopkins Bloomberg School of Public Health, Baltimore, Maryland: Katherine L. O’Brien (principal investigator [PI]), Orin S. Levine (former PI, current affiliation Bill & Melinda Gates Foundation, Seattle, Washington), Maria Deloria Knoll (co-PI), Daniel R. Feikin (joint affiliation with Centers for Disease Control and Prevention [CDC], Atlanta, Georgia), Andrea N. DeLuca, Amanda J. Driscoll, Nicholas Fancourt, Wei Fu, Laura L. Hammitt, Melissa M. Higdon, E. Wangeci Kagucia, Ruth A. Karron, Mengying Li, Daniel E. Park, Christine Prosperi, Zhenke Wu, Scott L. Zeger; Emmes Corporation, Rockville, Maryland: Nora L. Watson, Nuffield Department of Clinical Medicine, University of Oxford, United Kingdom: Jane Crawley; University of Otago, Christchurch, New Zealand: David R. Murdoch; icddr,b, Dhaka and Matlab, Bangladesh: W. Abdullah Brooks (site PI), Hubert P. Endtz, Khalequ Zaman, Doli Goswami, Lokman Hossain, Yasmin Jahan, Hasan Ashraf; Medical Research Council, Basse, The Gambia: Stephen R. C. Howie (site PI), Bernard E. Ebruke, Martin Antonio, Jessica McLellan, Eunice Machuka, Arifin Shamsul, Syed M.A. Zaman, Grant Mackenzie; KEMRI–Wellcome Trust Research Programme, Kilifi, Kenya: J. Anthony G. Scott (site PI and PERCH co-PI), Juliet O. Awori, Susan C. Morpeth, Alice Kamau, Sidi Kazungu, Micah Silaba Ominde; Division of Infectious Disease and Tropical Pediatrics, Department of Pediatrics, Center for Vaccine Development, Institute of Global Health, University of Maryland School of Medicine, Baltimore, Maryland and Centre pour le Développement des Vaccins (CVD-Mali), Bamako, Mali: Karen L. Kotloff (site PI), Milagritos D. Tapia, Samba O. Sow, Mamadou Sylla, Boubou Tamboura, Uma Onwuchekwa, Nana Kourouma, Aliou Toure; Respiratory and Meningeal Pathogens Research Unit, University of the Witwatersrand, Johannesburg, South Africa: Shabir A. Madhi (site PI), David P. Moore, Peter V. Adrian, Vicky L. Baillie, Locadiah Kuwanda, Azwifarwi Mudau, Michelle J. Groome, Nasreen Mahomed; Thailand Ministry of Public Health–US CDC Collaboration, Nonthaburi, Thailand: Henry C. Baggett (site PI), Somsak Thamthitiwat, Susan A. Maloney (former site PI), Charatdao Bunthi, Julia Rhodes, Pongpun Sawatwong, Pasakorn Akarasewi (site co-PI, Ministry of Public Health); Boston University School of Public Health, Boston, Massachusetts and University Teaching Hospital, Lusaka, Zambia: Donald M. Thea (site PI), Lawrence Mwananyanda, James Chipeta, Phil Seidenberg, James Mwansa, Somwe wa Somwe, Geoffrey Kwenda; Canterbury Health Laboratory, Christchurch, New Zealand: Trevor P. Anderson, Joanne Mitchell.

***Financial support.*** PERCH was supported by the Bill & Melinda Gates Foundation (grant number 48968 to the International Vaccine Access Center, Department of International Health, Johns Hopkins Bloomberg School of Public Health). J. A. G. S. was supported by a clinical fellowship from the Wellcome Trust of Great Britain (award number 098532).

***Supplement sponsorship.*** This article appears as part of the supplement “Pneumonia Etiology Research for Child Health (PERCH): Foundational Basis for the Primary Etiology Results,” sponsored by a grant from the Bill & Melinda Gates Foundation to the PERCH study of Johns Hopkins Bloomberg School of Public Health, Baltimore, Maryland.

***Potential conflicts of interest.*** K. L. O. has received grant funding from GSK and Pfizer and participates on technical advisory boards for Merck, Sanofi Pasteur, PATH, Affinivax, and ClearPath. M. D. K. has received funding for consultancies from Merck, Pfizer, and Novartis, and grant funding from Merck. L. L. H. has received grant funding from Pfizer and GlaxoSmithKline. K. L. K. has received grant funding from Merck Sharp & Dohme. S. A. M. has received honoraria for advisory board membership from the Bill & Melinda Gates Foundation, Pfizer, Medimmune, and Novartis; has received institutional grants from GSK, Novartis, Pfizer, Minervax, and the Bill & Melinda Gates Foundation; and has served on speaker’s bureaus for Sanofi Pasteur and GSK. All other authors report no potential conflicts. All authors have submitted the ICMJE Form for Disclosure of Potential Conflicts of Interest. Conflicts that the editors consider relevant to the content of the manuscript have been disclosed.
