# Supplementary material for: Colonization Density of the Upper Respiratory Tract as a Predictor of Pneumonia—Haemophilus influenzae, Moraxella catarrhalis, Staphylococcus aureus, and Pneumocystis jirovecii
Source: Clin Infect Dis. 2017 May 27;64(Suppl 3):S328–36. doi: 10.1093/cid/cix104 (PMC5612712; doi:10.1093/cid/cix104)
Supplement: Supplemental Materials [file cix104_suppl_supplemental_materials.docx]

Supplemental Figure 1. Density distributions^a^ between confirmed cases, radiographic pneumonia (CXR+) cases^b^ and controls


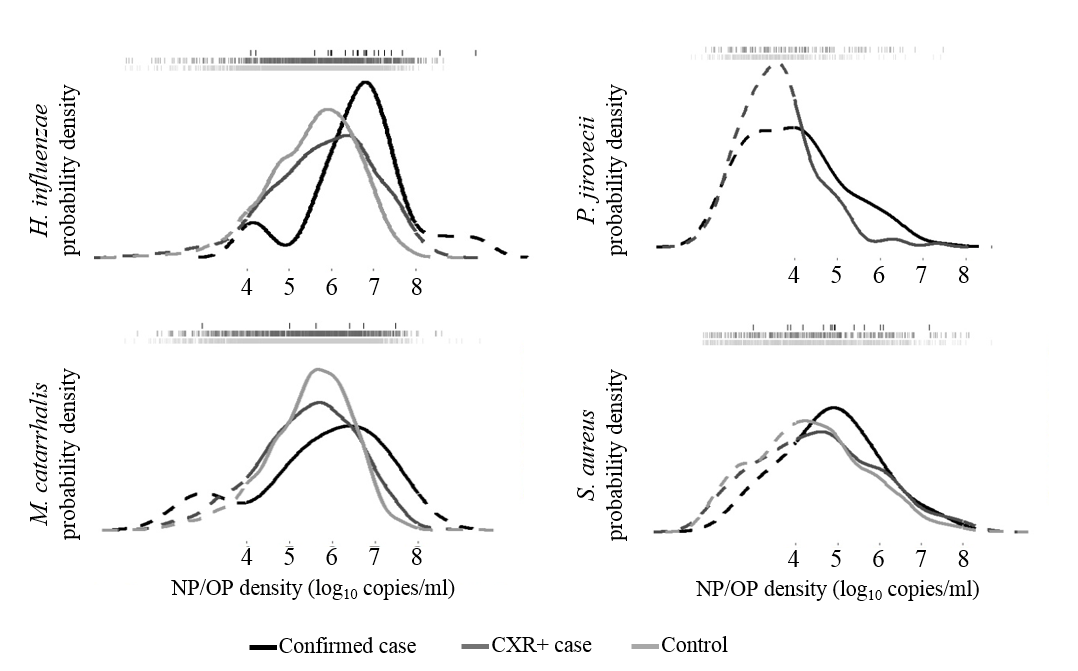


^a^ Restricted to children positive on nasopharyngeal/oropharyngeal PCR for the given pathogen.

^b^ Defined as having radiographic evidence of pneumonia (consolidation and/or other infiltrate).

Supplemental Figure 2. Colonization density (log_10_ copies/mL)^a^ among confirmed cases, radiographic pneumonia (CXR+) cases^b^ without microbiologically confirmed pathogens, and controls, by site and case status

**
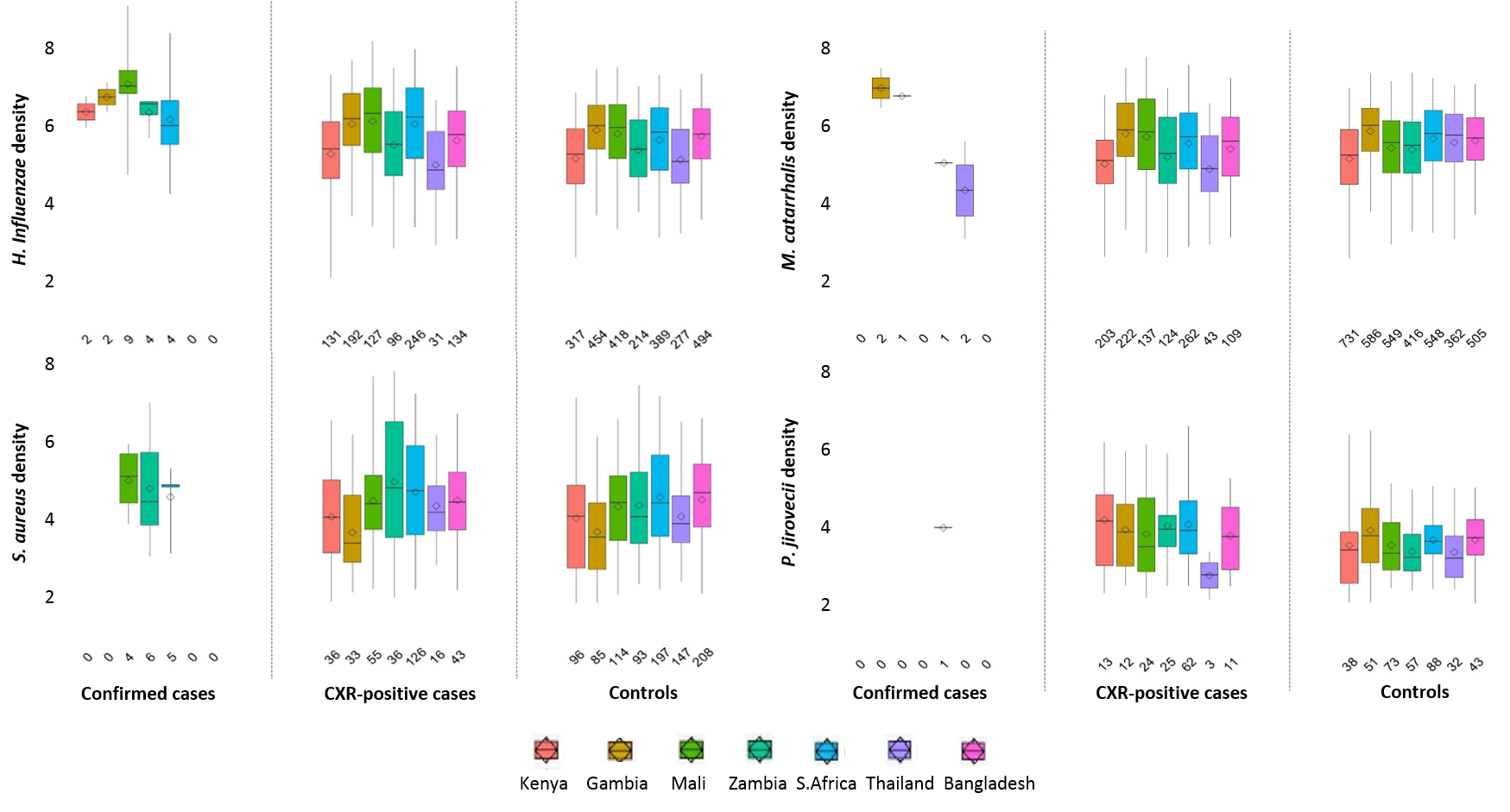
**

Diamonds are group means. Horizontal lines through boxes are group medians. Boxes extend to the 25th and 75th percentiles. Whiskers represent the 95% confidence interval. Numbers below the boxplots represent the number of positive cases.

^a^ Restricted to children positive on NP/OP for the given pathogen.

^b^ CXR-positive defined as having radiographic evidence of pneumonia (consolidation and/or other infiltrate).

Supplemental Figure 3. Colonization densities^a^ among radiologic pneumonia (CXR-positive) cases^b^ and controls, by prior antibiotics and case status


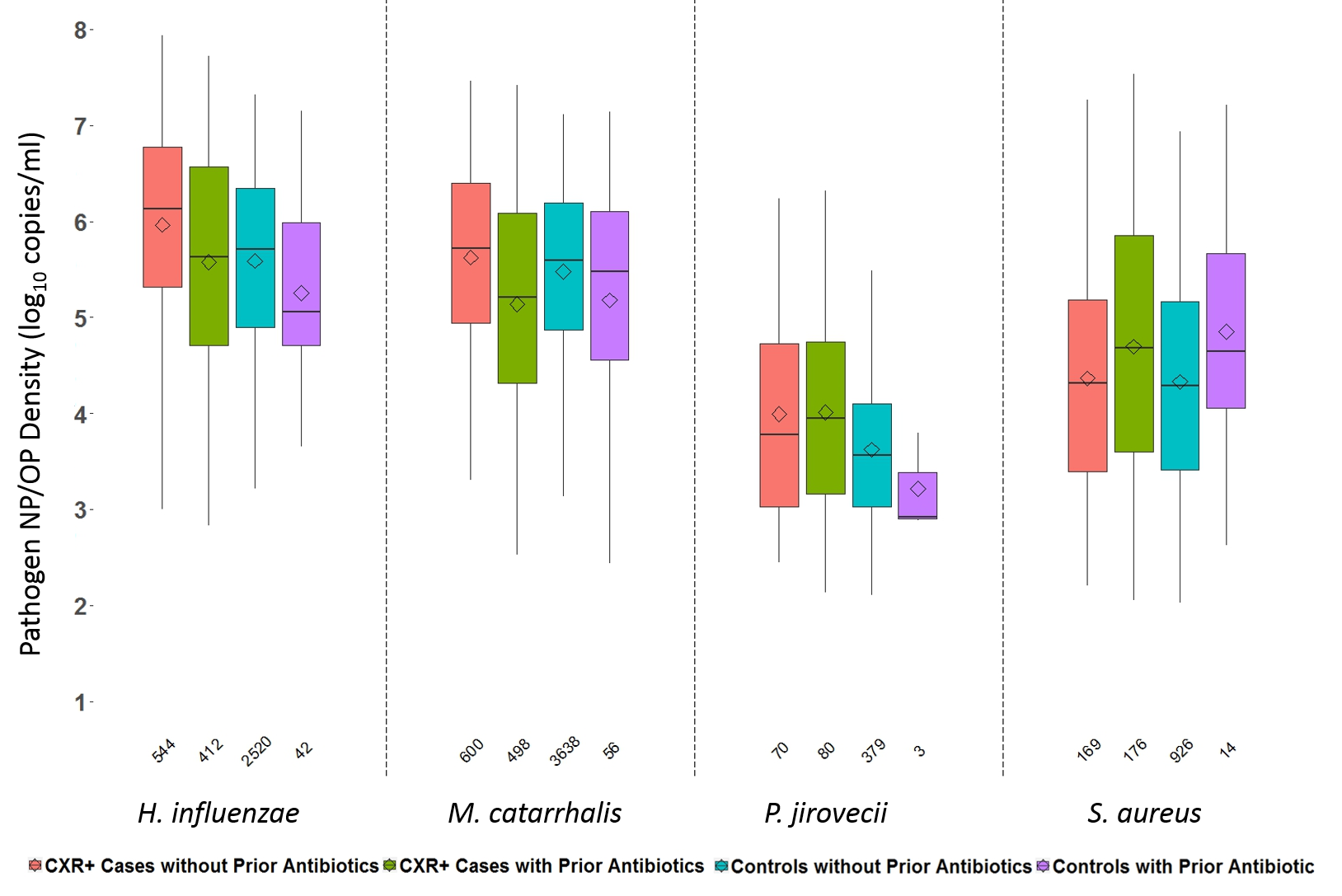


Diamonds = group means. Horizontal lines through boxes = group medians. Boxes extend to the 25th and 75th percentiles. Whiskers represent the 95% confidence interval. Numbers above the pathogen names represent the number of positive cases.

^a^ Figures restricted to children positive on NP/OP for the given pathogen.

^b^ CXR-positive defined as having radiographic evidence of pneumonia (consolidation and/or other infiltrate).

Supplemental Figure 4. Kernel density distribution plot^a^ of *P. jirovecii* density among radiographic pneumonia (CXR+) cases^b^ and all controls by HIV infection status


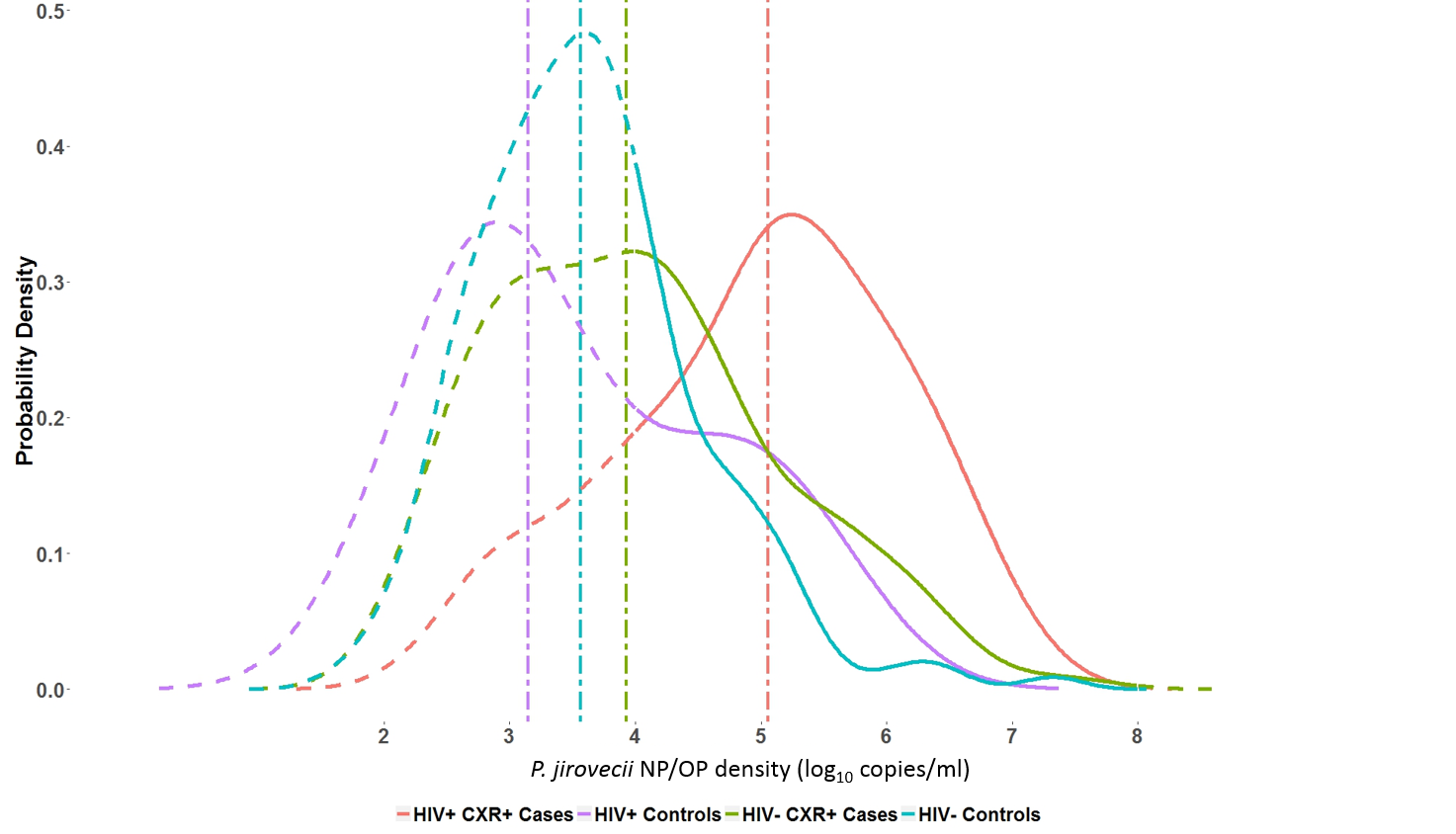


^a^ Figure restricted to children positive on NP/OP for the given pathogen. Restricted to children enrolled at the South Africa or Zambia sites.

^b^ CXR-positive defined as having radiographic evidence of pneumonia (consolidation and/or other infiltrate).

Supplemental Figure 5. Receiver operating characteristic (ROC) curve and Youden index analysis^a^, radiographic pneumonia cases^b^ compared with controls

**
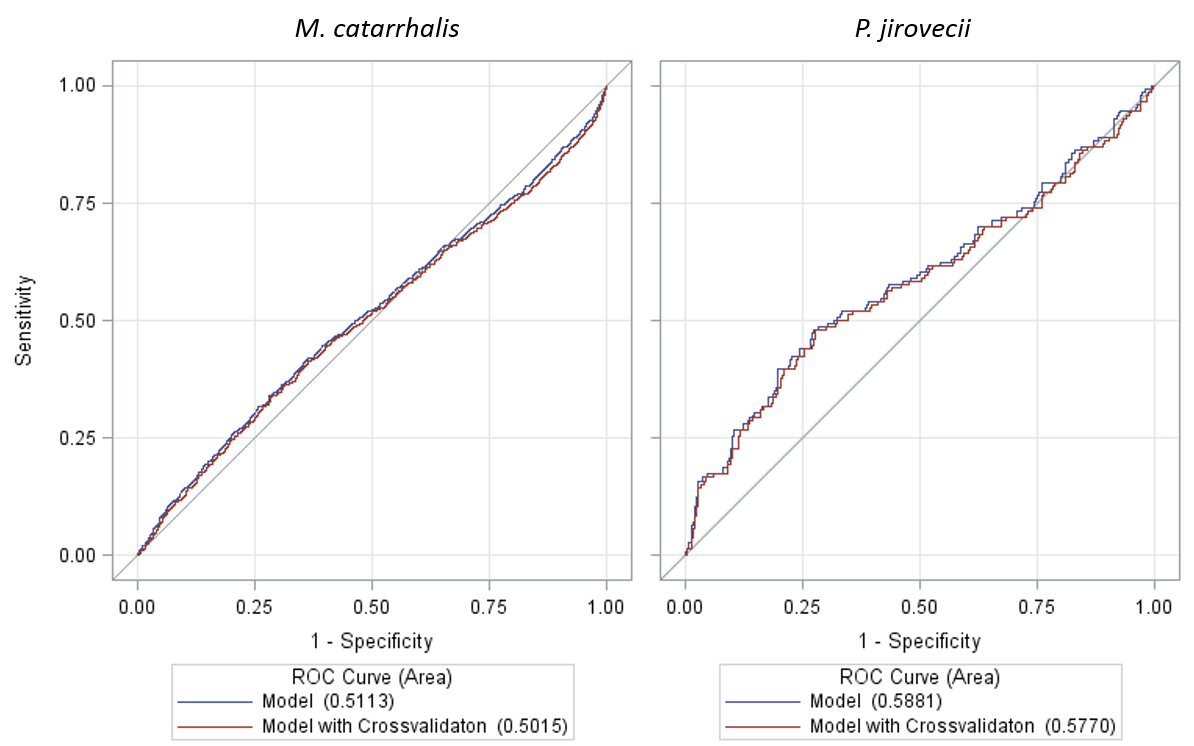
**

^a^ Analysis restricted to children positive on nasopharynx/oropharynx PCR.

^b^ CXR-positive defined as having radiographic evidence of pneumonia (consolidation and/or other infiltrate).

Supplemental Figure 6. Descriptive display of proportional bacterial^a^ colonization density and *M. catarrhalis* dominated profiles (grey shading) detected in the nasopharynx/oropharynx by PCR among 100 randomly selected controls and confirmed *H.influenzae, M. catarrhalis, S. aureus,* and *S. pneumoniae* cases.

**
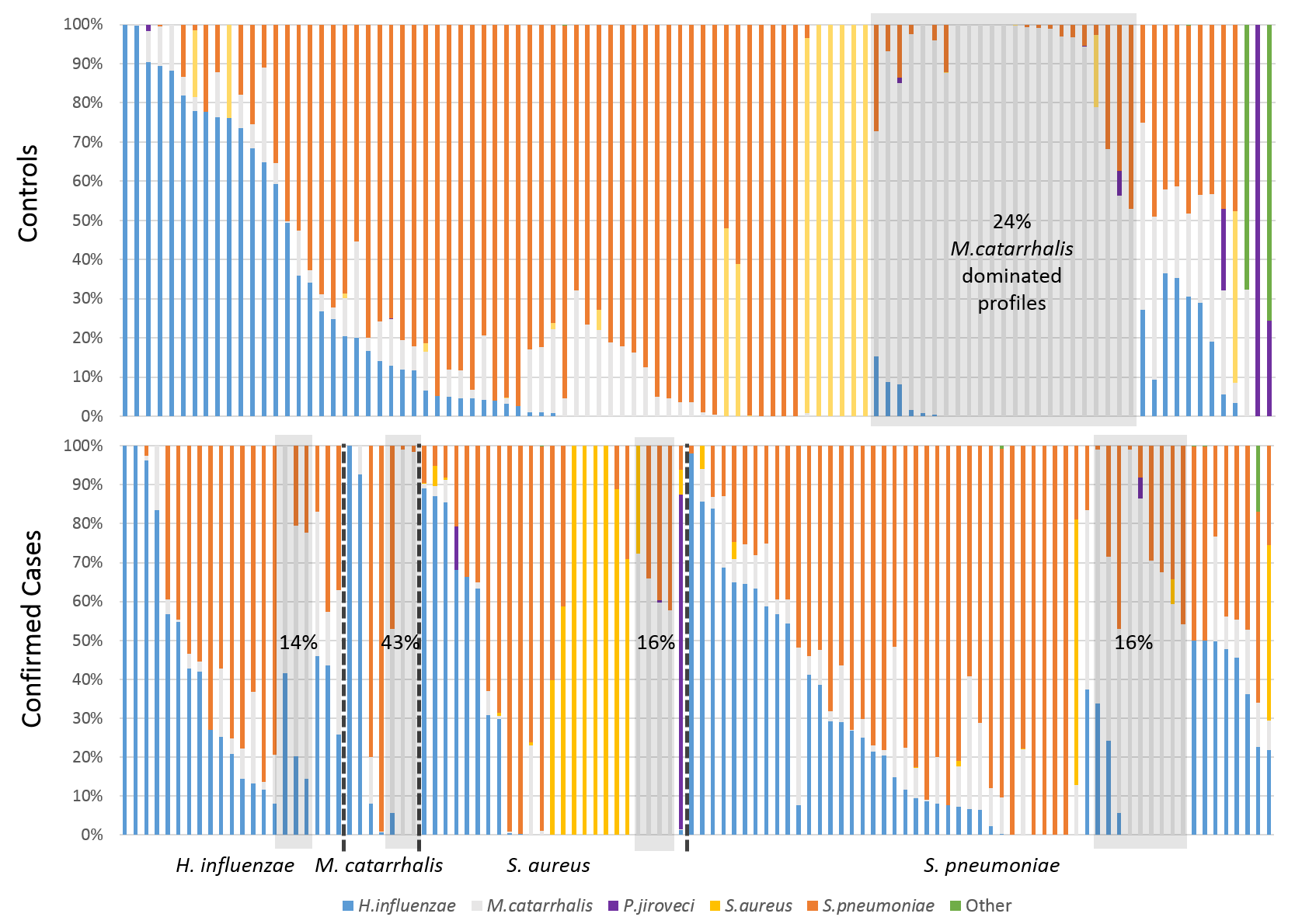
**

^a^ Including *B. pertussis, C. pneumoniae, H. influenzae, M. catarrhalis, M. pneumoniae, P. jirovecii, S. aureus, Salmonella* species, and *S. pneumoniae*.

**Acknowledgements:**

***PERCH Expert Group.*** William C. Blackwelder, Harry Campbell, John A. Crump, Adegoke Falade, Menno D. de Jong, Claudio Lanata, Kim Mulholland, Shamim Qazi, Cynthia G. Whitney.

***Pneumonia Methods Working Group.*** Robert E Black, Zulfiqar A Bhutta, Harry Campbell, Thomas Cherian, Derrick W Crook, Menno D de Jong, Scott F Dowell, Stephen M Graham, Keith P Klugman, Claudio F Lanata, Shabir A Madhi, Paul Martin, James P Nataro, Franco M Piazza, Shamim A Qazi, and Heather J Zar.

***PERCH Chest Radiograph Reading Panel***

**Readers:** Dr. Kamrun Nahar, Dr. Fariha Bushra Matin, Dr. Claire Oluwalana, Dr. Bernard Ebruke, Dr. Joyce Sande, Dr. Micah Silaba Ominde, Dr. Mahamadou Diallo, Dr. Breanna Barger-Kamate, Dr. Nasreen Mahomed, Dr. David P. Moore, Dr. Anchalee Kruatrachue, Dr. Piyarat Suntarattiwong, Dr. Musaku Mwenechanya, Dr. Rasa Izadnegahdar, **Arbitrators:** Dr. Vera Manduku, Dr. John DeCampo, Dr. Marg DeCampo, Dr. Fergus Gleeson.

***PERCH Contributors:***

**Bangladesh:** Kamrun Nahar, Arif Uddin Sikdir, Sharifa Yeasmin, Dilruba Ahmed, Muhammad Ziaur Rahman, Muhammad Yunus, Muhammad Al Fazl Khan, Muhammad Jubayer Chisti, Abu Sadat Muhammad Sayeem, Shahriar Bin Elahi, Mustafizur Rahman; **The Gambia:** Michel Dione, Emmanuel Olutunde, Peter Githua, Ogochukwu Ofordile, Rasheed Salaudeen, David Parker; **Kenya:** Shebe Mohamed, Siti Ndaa, Micah Silaba, Neema Muturi, Angela Karani, Sammy Nyongesa, Anne Bett, Daisy Mugo, Salim Mwarumba, Robert Musyimi, Andrew Brent, James Nokes, David Mulewa, Joyce Sande, John Odhiambo, Joshua Wambua, Nuru Kibirige, Caroline Mulunda, Hellen Mjalla, Norbert Katira, Karen Dama, Loice Masha, Christine Mutunga, Mwanajuma Ngama, Stephen Mangi, Riziki Anthony, Mwarua Yubu, Elijah Wakili, Benson Katana, Shoboi Mgunya, Emmanuel Mumba, Benedict Mver, George Kuria, Felix Githinji, Norbert Kihuha, Boniface Jibendi, Tahreni Bwanaali, Agustus Kea; **Mali:** Nana Kourouma, Aliou Toure, Mahamadou Diallo, Breana Barger-Kamate, Mariam Samake, Seydou Sissoko, Abdoul Aziz Maiga, Mariam Samake, Toumani Sidibe, Mariam Sylla, Aziz Diakite, Bassirou Diarra; **South Africa:** Azwidihwi Takalani, Andrea Hugo, Susan Nzenze, Ndulela Titi, Mmabatho Selela, Malebo Motiane, Minah Nkuna, Nonhlanhla Tsholetsane, Sibonsile Moya, Debra Katisi, Tondani Netshishivhe, Lerato Mapetla, Gudani Singo, Simphiwe Gasa, Cece Mgenge, Nozipho Mthunzi, Nombulelo Monedi, Tanja Adams, Shafeeka Mangera, Jeannette Wadula, Peter Tsaagane, Jenifer L. Vaughan, Sakina Loonat, Martin Hale, Sugeshnee Pather, Mariëtte Middel, Siobhan Trenor, Palesa Morailane, Ntombi Maya, Rene Sterley, Charné Combrinck, Given Malete, Lerato Qoza, Grizelda Liebenberg, Hendrik van Jaarsveld, Zunaid Kraft, Lisa-Marie Mollentze, Lourens Combrinck, Tsholofelo Mosome; **Thailand:** Sununta Henchaichon, Dr. Tussanee Amornintapichet, Dr. Somchai Chuananont, Toni Whistler, Juraiporn Ratanodom, Patranuch Sapchookul, Ornuma Sangwichian, Sirirat Makprasert, Manoon Hirunsalee, Possawat Jorakate, Anek Kaewpan, Duangkamol Siludjai, Apiwat Lapamnouysup, Dr. Wantana Paveenkittiporn, Waraporn Ubonphen, Dr. Peera Areerat, Ms.Yupapan Wannachaiwong, Ms Tewa Faipet, Ms Punnat Natnarakorn, Ms Ahchanan Sacharone, Mr.Winai Makmool, Ms. Kanlaya Sornwong, Ms. Promporn Sansuriwong, Ms. Ratchanida Potiya, Ms. Wasana Hongsawong, Ms.Wipa Matchaikhen, Ms. Thatsanawan Chaiyabil, Ms.Piyapai Wannarach, Ms Chamaiporn Wadeesirisak, Mr. Yuttapong Norapet, Mattana Bangkung, Mr. Barameht Piralam, Sathapana Naorat, Anchalee Jatapai, Prasong Srisaengchai, Dr. Leonard Peruski, Ms.Dawan Phaensoongnoen, Ms.Tussaaorn Klangprapan, Ms.Narawadee Dumrongdee, Ms.Atchara Srithongkham, Mr. Piyawut Noinont, Ms. Pornthip Kamlee, Ms.Siyapa Mongkornsuk; **Zambia:** Justin Mulindwa, Musaku Mwenechanya, John Mwaba, Magdalene Mwale, Julie Duncan, Kazungu Siazele, Muntanga Mapeni, Emily Hammond; **Canterbury Health Laboratory, Christchurch, New Zealand:** Rose Watt, Shalika Jayawardena; **The Emmes Corporation, Rockville, Maryland:** Mark Wolff, Megan Sanza, Omid Neyzari.
